# P04.18. Current status of the dual medical license holders in Korea

**DOI:** 10.1186/1472-6882-12-S1-P288

**Published:** 2012-06-12

**Authors:** B Choi, G Han, B Lim, H Cho

**Affiliations:** 1 Pusan National University School of Korean Medicine, Yangsan, Gyeongnam, Republic of Korea; 2 Department of Health Policy and Management, Sangji University, Wonju, Republic of Korea

## Purpose

Korea’s medical system, comprising of conventional western medicine (CWM) and traditional Korean medicine (TKM), is at the same time dichotomistic and dualistic. TKM has its own systems for education and national licensing. While there is a sharp distinction between the practices of medical doctors (MD) and TKM doctors (TKMD) legally, there are also many conflicts arising from crossover practices in reality. Dual medical license holders (DMD) having both MD and TKMD licenses are increasing because of this contradictory situation. This study aims to investigate the status of DMDs and provide basic data for developing strategies of cooperation between CWM and TKM.

## Methods

The questionnaires on general characteristics and working status were developed and administered to both DMDs and medical students pursing second medical license in order to become DMDs. The data from 121 DMDs and 61 students were collected with the help of the Association of DMDs and analyzed statistically.

## Results

Since 1996 the number of DMDs has been increasing rapidly and reached 206 in 2010, according to official records. This figure represents less than 1% of TKMDs and less than 0.2% of MDs. The mean age of DMDs is 42.28±6.54 years, and most of them are male (86.0%). Seventy-five percent of DMDs obtained the MD license first. These ratios are reversed among the students, of whom 73.7% are KMD. The mean time for obtaining the additional license was 10.11±4.91 years. Since the revision of Korean medical law in 2009, DMDs can open both medical clinics and Korean medicine clinics. However, only 41.9% of DMDs are opening both clinics.

## Conclusion

It can be expected that more KMDs will choose to become DMDs in the future, and the number of DMDs as well as their role in medical collaboration will continue to expand. To promote this process, developing an integrative medical curriculum should be enhanced.

